# Infusional 5-fluorouracil, cisplatin and mitomycin C in advanced gastric cancer : A low cost effective regimen

**DOI:** 10.1038/sj.bjc.6600046

**Published:** 2002-01-21

**Authors:** S Cascinu, A M Baldelli, V Catalano, P Giordani, G D Beretta, R R Silva, G Gasparini, D Mari, R Maisano, S Salvagni, S Barni, R Labianca, L Frontini, C Curti, G Catalano

**Affiliations:** Department of Medical Oncology, V.le Gramsci 14, 43100 Parma, Italy; Department of Medical Oncology, Azienda Osp. S. Salvatore, 61100 Pesaro, Italy; Department of Medical Oncology, Ospedali Riuniti, L.go Barozzi 1, 24128 Bergamo, Italy; Department of Medical Oncology, Ospedali E. Profili, 60044 Fabriano (AN), Italy; Department of Medical Oncology, Azienda Osp. S. Filippo Neri, 00135 Rome, Italy; Department of Medical Oncology, University of Messina, 98123 Messina, Italy; Department of Medical Oncology, Azienda Osp. Treviglio-Caravaggio, P.le Ospedale 1, 24047 Treviglio (BG), Italy; Department of Medical Oncology, S. Paolo Hospital, 20100 Milan, Italy; Coordinating Data Center GISCAD, 20100 Milan, Italy

**Keywords:** chemotherapy, metastatic gastric cancer, infusional 5-FU, cisplatin

## Abstract

Recently, we reported a highly active regimen in advanced gastric cancer including a weekly administration of cisplatin, epidoxorubicin, leucovorin, 5-fluorouracil with the support of filgrastim. In order to simplify the administration and to decrease the toxicity of these drugs, mainly epidoxorubicin-induced alopecia, we designed a regimen including an infusional 5-fluorouracil schedule according to the de Gramont regimen, cisplatin and mitomycin C replacing epidoxorubicin. Forty-five patients with advanced or metastatic gastric cancer were treated with cisplatin 50 mg m^−2^ i.v. on day 1, every 2 weeks, 6S-stereoisomer-leucovorin 100 mg m^−2^ i.v. followed by 5-fluorouracil 400 mg m^−2^ i.v. bolus and 600 mg m^−2^ i.v. in a 22-h infusion, on days 1 and 2, every 2 weeks, and mitomycin C 7 mg m^−2^ i.v. bolus on day 2, every 6 weeks. Grades 3–4 toxicities (National Cancer Institute-Common Toxicity Criteria) consisted mainly of neutropenia and thrombocytopenia. Five patients had a complete response and 16 had a partial response for an overall response rate of 46.7% (95% confidence interval, 32.1–61.2%). The median survival was 11 months. The combination of cisplatin, 5-fluorouracil and leucovorin according to de Gramont, and mitomycin C seems to be an active and safe regimen in the treatment of advanced gastric cancer. Because of its low cost it may be suggested for patients not enrolled into clinical trials.

*British Journal of Cancer* (2002) **86**, 213–217. DOI: 10.1038/sj/bjc/6600046 www.bjcancer.com

© 2002 The Cancer Research Campaign

## 

Although several new chemotherapeutic drugs have been recently introduced into clinical practice, metastatic gastric carcinoma still remains an incurable disease with poor median survival ranging from 6 to 8 months (Schipper and Wagener, 1996). During the last decade, various randomized trials have demonstrated the improvement of 5-fluorouracil (5-FU)-based regimens in terms of overall survival and quality of life in patients with advanced gastric cancer in comparison to the best supportive care (Murad *et al*, 1993; Pyrhonen *et al*, 1995; Glimelius *et al*, 1997). Moreover, in phase II studies the combination of cisplatin (CDDP) and 5-FU showed promising results both in the metastatic disease (Lacave *et al*, 1991), and in the preoperative setting (Rougier *et al*, 1994). In advanced gastric cancer, two phase III randomized studies compared the efficacy of a combination of CDDP and infusional 5-FU (FP) to other 5-FU-based chemotherapies. In the first study (Kim *et al*, 1993), FP achieved a significantly higher response rate and a longer time to progression than 5-FU, doxorubicin (ADR), and mitomycin C (MMC) (FAM regimen) or infusional 5-FU, but survival was not significantly ameliorated. In the other study, Vanhoefer *et al* (2000) compared the efficacy and tolerability of FP or etoposide, folinic acid, and 5-FU (ELF) with that of the standard 5-FU, ADR, and sequential high-dose methotrexate (FAMTX). However, all three treatments showed modest clinical activity and comparable toxicity, neutropenia being the most frequent side-effect.

In 1997, we reported interesting results in the treatment of advanced gastric cancer with an intensive weekly low dose 5-FU, epidoxorubicin (epiADR), CDDP, 6S-stereoisomer-leucovorin (LV), and bone marrow support with the haemopoietic growth factor filgrastim (Cascinu *et al*, 1997). The choice of these drugs and schedule was made based on the following: 5-FU, epiADR and CDDP remain the most active single agents in gastric cancer (Schipper and Wagener, 1996); 5-FU and CDDP are potentially synergistic (Leichman and Berry, 1991); LV can enhance 5-FU activity (Arbuck *et al*, 1987); weekly administration allows more drug to be administered per unit time, which minimizes side effects (Young, 1990); filgrastim may contribute to perform weekly administrations of drugs reducing the incidence of neutropenia. This weekly intensive regimen confirmed its activity also in locally advanced gastric cancer enabling resection on half of previously inoperable tumour with a moderate toxicity (Cascinu *et al*, 1998), and now a randomized trial in the adjuvant setting is being carried out in Italy.

A potential drawback of this weekly regimen may be the economic cost of filgrastim administration. While in preoperative chemotherapy or in the adjuvant setting it may be justifiable, it may not be cost-effective in the advanced disease. Furthermore, in our regimen the schedule of 5-FU given by bolus injection may not be optimal. The combination of 5-FU and LV according to de Gramont allows for the administration of higher doses of 5-FU; furthermore, the continuous infusion increases the time exposition of tumour cells to 5-FU, which may translate into a better oncolytic effect (de Gramont *et al*, 1997).

Based on these considerations and on the results of a bi-weekly infusional administration of 5-FU, according to de de Gramont *et al* (1997), we tested a new combination associating CDDP and MMC to the de Gramont regimen. While the contribution of epiADR to the combination of 5-FU and CDDP is unclear, MMC has been reported to be one of the most active cytotoxic drugs in this disease (Schnall and Macdonald, 1993). Another peculiar aspect of MMC is the lower incidence of alopecia than that induced by epiADR. Herein, we reported the results of a multicentre phase II study in order to assess safety and efficacy of bi-weekly CDDP and de Gramont regimen in combination with MMC.

## PATIENTS AND METHODS

### Patient selection

Patients with histologically confirmed unresectable locally advanced and/or metastatic gastric carcinoma were eligible for the study. Patients thought to have potentially curable disease by resection of the primary tumour were not eligible. Patients were required to have measurable disease, defined as the presence of lesion identified bidimensionally by CT scan. Patients with nonmeasurable disease as the only reference were not included, and in the case of radiotherapy to individual sites of disease, they were not considered evaluable for response. Other eligibility criteria were age ⩽70 years, Eastern Cooperative Oncology Group (ECOG) performance status 0 to 2, and normal liver (serum bilirubin <1.5 mg dl^−1^), renal (serum creatinine <1.5 mg dl^−1^) and bone marrow (neutrophils >2×10^9^ l^−1^, platelets >100×10^9^ l^−1^) functions. Informed consent was obtained from all participants after the nature of the study had been fully explained. The protocol was approved by the local ethic committees.

### Chemotherapy

The treatment schedule consisted of CDDP 50 mg m^−2^ as a 30-min infusion, on day 1; LV 100 mg m^−2^ diluted in 250 ml of normal saline solution in a 2-h infusion followed by 5-FU 400 mg m^−2^ i.v. bolus and 5-FU 600 mg m^−2^ in a 22-h infusion, repeated for 2 consecutive days; MMC 10 mg m^−2^ as a bolus injection, on day 2, every 42 days. Subsequent to the first 10 patients developing severe toxicity, the planning dose of MMC was reduced to 7 mg m^−2^ (cumulative total dose of 28 mg m^−2^, maximum 56 mg), as suggested previously by Ross *et al* (1997). Standard intravenous hydration was used: 2 h before initiation of the CDDP infusion, patients received 1500 ml of 0.9% sodium chloride to which 20 mequiv of potassium chloride and 15 mequiv of magnesium sulphate were added. Post-hydration was continued for 2 h with 1000 ml of normal saline solution. As antiemetic regimen, all patients received dexamethasone 20 mg in 50 ml of saline solution given as an intravenous infusion over 15 min, 45 min before CDDP, and 5HT-3 antagonists (ondansetron 8 mg or tropisetron 5 mg) in 50 ml of normal saline as intravenous infusion over 15 min. Treatment was repeated every 14 days for a minimum of six cycles and stopped in case of unacceptable toxicity, disease progression, or patient refusal. Treatment was administered through a central venous line, or a venous totally implantable port, connected with external pumps.

### Evaluation of toxicity and response

Chemotherapy toxicity was assessed every 2 weeks according to the National Cancer Institute Common Toxicity Criteria (National Cancer Institute–CTC) (1990). Patients were to be removed from the study for any treatment delay longer than 3 weeks. Chemotherapy was delayed until recovery if neutrophils decreased to ⩽1.5×10^9^ l^−1^, or platelet count decreased to ⩽100×10^9^ l^−1^. A 25% dose reduction of chemotherapeutic drugs was mandatory in case of grade 4 neutropenia, grade 3–4 thrombocytopenia, grade 2–3 mucositis, diarrhoea, or hand-foot syndrome. Treatment was stopped in case of grade 4 mucositis, diarrhoea, or hand-foot syndrome. In the presence of other grade 4 NCI–CTC toxicities, patients should be withdrawn from the study. Tumour response was measured according to the World Health Organization (WHO) criteria, and was performed after six and 12 cycles of therapy. Histologic confirmation at endoscopy of tumoural resolution was required to determine a complete response at the primary site.

### Statistical methods

This was a multicentre phase II study. The primary end-points were to determine the response rate and toxicity. Secondary objectives were to measure the duration of response and survival. This study followed the minimax two-stage phase II design. The treatment program was designed to reject a response rate less than 20% (p0) and to provide a statistical power of 90% in assessing the activity of the regimen (in terms of response rate) as 40% (p1) (p1–p0=20%) for an alpha error less than 0.10 (Simon, 1989). The 95% exact confidence interval (CI) for response was calculated. Survival time was calculated from the onset of chemotherapy until death or the last visit for patients alive. Duration of response was calculated from the date of response to progression or death. Patients survival was examined using the Kaplan-Meier product limit method (Kaplan and Meier, 1958).

## RESULTS

Forty-five consecutive patients were entered into the study. At May 2001, the median follow-up duration from the start of treatment was 18 months (range 12 to 23 months). The characteristics of the patients are summarized in Table 1

**Table 1:**
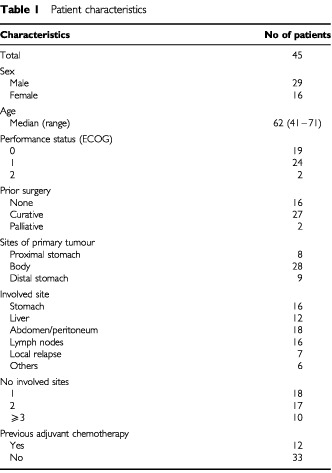
Patient characteristics

. Only three patients had locally advanced disease (two primary gastric cancers and one anastomotic relapse).

### Toxicity

A total of 330 cycles was delivered and the median number of cycles for patient who received treatment was six (range, 1 to 13). Seventeen patients received chemotherapy without any modification of the planned dose; in the other 28 patients treatment was modified due to toxicity (mainly, haematological). All 45 patients were evaluable for toxicity (Table 2

**Table 2:**
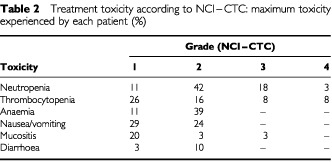
Treatment toxicity according to NCI–CTC: maximum toxicity experienced by each patient (%)

). Grade 3–4 neutropenia occurred in 21%, thrombocytopenia in 16%, and mucositis in 3% of patients. Other toxicities were mainly mild: nausea/vomiting, mucositis, diarrhoea. Grade 1–2 alopecia occurred in 26% of patients. No patient had treatment related death.

### Response and survival

Objective tumour response was observed in 21 of 45 patients for a response rate of 46.7% (95% CI, 32.1–61.2%) (Table 3

**Table 3:**
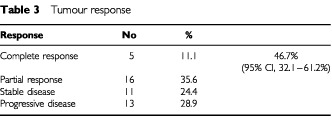
Tumour response

). Five patients achieved a complete response and 16 a partial response. The median duration of response was 7 months (range, 3–10). The response rate was not significantly affected by the number of metastatic sites (1 *vs* 2), nor by the type of metastases. Eleven patients had stable disease, whereas in 13 patients disease progressed while on therapy. The median survival of all patients was 11 months, with 1 and 2 year survivals of 45 and 10%, respectively.

## DISCUSSION

Our three drugs combination is a well tolerated and effective palliative chemotherapeutic regimen, and compares well with our previous intensive weekly regimen (Cascinu *et al*, 1997) in terms of activity, with 46% of response rate and a median survival of 11 months. Although the non randomized design of this study suggests caution in the interpretation of results, the objective tumour response rate is within the confidence limits observed in the cohort of patients treated previously with the intensive weekly regimen (Cascinu *et al*, 1997). Wilke *et al* (1996) showed that infusional 5-FU can be safely combined with bi-weekly CDDP in advanced gastric cancer. More recently, Kretzschmar *et al* (2000) reported the results of a weekly high-dose infusional 5-FU combined with MMC. In 34 evaluable patients, they obtained 11 objective response (37%) with a median survival time of 7 months. The survival reported in the present study is comparable to that obtained with the most intensive regimen, and clearly better than that reported with the combination of 5-FU with CDDP or MMC alone (Schnall and Macdonald, 1993; Wilke *et al*, 1996; Kretzschmar *et al*, 2000). Recently, Mitry *et al* (2000) reported interesting results with a similar regimen (de Gramont or its simplified modification plus CDDP) to that adopted in our study, but without the administration of MMC. A response rate of 46.1 and of 55.6% was achieved in 13 patients with gastric cancer and nine patients with gastro-oesophageal junction cancer, respectively. In this trial, a severe thrombocytopenia was observed in only one patient out of 39 compared with 21% of patients in the present report, an effect likely to be due to the administration of MMC in our study. The comparable efficacy and better tolerability of a CDDP and de Gramont regimen without the addition of MMC may warrant further evaluation.

Recently, the Royal Marsden Hospital reported the results of a phase II study on oesophago-gastric cancer patients treated with CDDP, infusional 5-FU and epiADR (ECF regimen) (Findlay *et al*, 1994). The activity of this regimen (71% of response rate) and the acceptable toxicity contributed to launch a large randomized trial in which ECF was compared to the standard FAMTX. ECF proved to be significantly better than FAMTX in terms of overall response rate (45 *vs* 21%, respectively; *P*=0.0002), median survival (8.8 versus 5.7 months, respectively; *P*=0.0009), quality of life and cost-effectiveness (Webb *et al*, 1997). In a second randomized trial on advanced oesophago-gastric cancer patients, the ECF regimen was compared to MCF, with MMC used in substitution of epiADR. The preliminary results of the study showed similar activity with both MCF and ECF (Ross *et al*, 1999).

Although MMC has only limited single-agent activity in colorectal cancer, in this disease the addition of MMC to 5-FU improved the response rate and progression-free survival relative to 5-FU (Ross *et al*, 1997), suggesting the possibility of a clinical synergism between MMC and infusional 5-FU, similarly to that obtained with CDDP (Becker *et al*, 1997. Moreover, the use of MMC is correlated with a lower incidence of alopecia than that given by epiADR. If we consider that alopecia is a relevant psychosocial problem for cancer patients, and if MMC is similarly active than epiADR in gastric cancer, it would be advantageous for patients to receive a regimen including MMC instead of epiADR. Interestingly, in the study comparing ECF with MCF, quality of life was superior with ECF (Ross *et al*, 1999); but it is generally hard to understand how important on health status may be a single aspect like alopecia when the global health-functioning aspect is translated into a score.

New cytotoxic agents, such as irinotecan, paclitaxel and docetaxel could be a more appealing approach for the treatment of advanced gastric cancer (Kollmannsberger *et al*, 2000; Mavroudis *et al*, 2000; Roth *et al*, 2000). Docetaxel obtained a 20% response rate as first-line treatment in 37 advanced gastric patients (Sulkes *et al*, 1994) with a median survival time of 7 months. However, at the dose of 100 mg m^−2^ every 3 weeks, filgrastim had to be given for 7 days. When lower doses of docetaxel in a weekly schedule were attempted, no clinical relevant results were found (Graziano *et al*, 2000). Preliminary data on docetaxel (at a dose of 85 mg m^−2^) combined with CDDP every 3 weeks seem to be promising with a response rate of 56% (Roth *et al*, 2000). Similarly, Kollmannsberger *et al* (2000) reported a response rate of 51% combining paclitaxel with CDDP and 5-FU.

However, the results obtained with these newer schedules seem to be comparable with those of our regimen. New drugs such as docetaxel and paclitaxel may increase the economic cost of chemotherapy, as a consequence of the major cost of such drugs, of the more frequent incidence of severe toxicity, mainly haematological, which may require in most cases the preventive use of haemopoietic growth factors and the need of longer hospitalization.

In conclusion, our data show that 5-FU, MMC and CDDP can be safely administered in combination, and that such a combination may have a therapeutic activity, similar to more aggressive regimens, or regimens including new and much more expensive drugs. In metastatic gastric cancer patients not eligible for entry into clinical trials, this regimen may be a reasonable option. A multicentre cooperative group is now evaluating this regimen in a randomized trial on patients with advanced gastric cancer.
